# Supplementary material for: Hot spot prediction in protein-protein interactions by an ensemble system
Source: BMC Syst Biol. 2018 Dec 31;12(Suppl 9):132. doi: 10.1186/s12918-018-0665-8 (PMC6311905; doi:10.1186/s12918-018-0665-8)
Supplement: Supplementary file 2 — All relevant MATLAB code. A simple Matlab implement of our predictor. (ZIP 19,742 kb) [file 12918_2018_665_MOESM2_ESM.zip › Additional file 2/Read ASA/ASA Property/SKEMPI/1AHWC.txt]

 NetSurfP - Protein Surface Accessibility and Secondary Structure Predictions

  

|  |  |  |
| --- | --- | --- |
|  |  | NetSurfP - Protein Surface Accessibility and Secondary Structure Predictions  Technical University of Denmark |

  


---

```
# For publication of results, please cite:
# A generic method for assignment of reliability scores applied to solvent accessibility predictions.
# Bent Petersen, Thomas Nordahl Petersen, Pernille Andersen, Morten Nielsen and Claus Lundegaard
# BMC Structural Biology 2009, 9:51 doi:10.1186/1472-6807-9-51
#
# Column 1: Class assignment - B for buried or E for Exposed - Threshold: 25% exposure, but not based on RSA
# Column 2: Amino acid
# Column 3: Sequence name
# Column 4: Amino acid number
# Column 5: Relative Surface Accessibility - RSA
# Column 6: Absolute Surface Accessibility
# Column 7: Z-fit score for RSA prediction
# Column 8: Probability for Alpha-Helix
# Column 9: Probability for Beta-strand
# Column 10: Probability for Coil
E T  1AHWC                  1    0.825 114.372  -1.498   0.003   0.003   0.994
E N  1AHWC                  2    0.824 120.604  -0.622   0.005   0.015   0.979
B T  1AHWC                  3    0.280  38.850  -0.106   0.005   0.015   0.979
E V  1AHWC                  4    0.375  57.699  -0.214   0.005   0.015   0.979
E A  1AHWC                  5    0.498  54.858   0.824   0.018   0.019   0.964
B A  1AHWC                  6    0.157  17.268  -0.923   0.005   0.045   0.951
E Y  1AHWC                  7    0.443  94.626  -0.078   0.004   0.197   0.799
E N  1AHWC                  8    0.410  60.068   0.110   0.004   0.420   0.576
B L  1AHWC                  9    0.049   8.935   0.677   0.003   0.718   0.279
E T  1AHWC                 10    0.291  40.292   1.499   0.001   0.900   0.099
B W  1AHWC                 11    0.043  10.221   0.568   0.001   0.959   0.040
E K  1AHWC                 12    0.248  50.993   1.321   0.001   0.959   0.040
B S  1AHWC                 13    0.036   4.172  -1.733   0.001   0.959   0.040
E T  1AHWC                 14    0.273  37.810   0.340   0.001   0.900   0.099
E N  1AHWC                 15    0.318  46.541  -1.413   0.005   0.262   0.733
B F  1AHWC                 16    0.192  38.615  -0.544   0.004   0.085   0.910
E K  1AHWC                 17    0.401  82.527   0.281   0.004   0.514   0.481
B T  1AHWC                 18    0.095  13.149   0.500   0.001   0.900   0.099
B I  1AHWC                 19    0.106  19.573   1.153   0.001   0.959   0.040
B L  1AHWC                 20    0.023   4.248   1.073   0.001   0.959   0.040
B E  1AHWC                 21    0.209  36.530   0.976   0.001   0.959   0.040
B W  1AHWC                 22    0.037   8.995  -0.488   0.001   0.900   0.099
E E  1AHWC                 23    0.419  73.112   0.942   0.004   0.514   0.481
B P  1AHWC                 24    0.247  35.007  -0.783   0.004   0.197   0.799
E K  1AHWC                 25    0.503 103.549   0.146   0.005   0.045   0.951
E P  1AHWC                 26    0.575  81.564  -0.982   0.003   0.003   0.994
E V  1AHWC                 27    0.693 106.576  -0.804   0.005   0.015   0.979
E N  1AHWC                 28    0.454  66.392  -0.973   0.004   0.138   0.858
B Q  1AHWC                 29    0.206  36.720   0.481   0.003   0.718   0.279
B V  1AHWC                 30    0.207  31.877   0.953   0.001   0.959   0.040
B Y  1AHWC                 31    0.064  13.698   0.662   0.001   0.959   0.040
B T  1AHWC                 32    0.140  19.432   0.973   0.001   0.959   0.040
B V  1AHWC                 33    0.034   5.164   0.731   0.001   0.959   0.040
B Q  1AHWC                 34    0.113  20.218   0.499   0.001   0.959   0.040
B I  1AHWC                 35    0.091  16.835   0.883   0.001   0.959   0.040
B S  1AHWC                 36    0.234  27.425   0.301   0.001   0.959   0.040
E T  1AHWC                 37    0.404  55.979  -0.434   0.004   0.616   0.381
E K  1AHWC                 38    0.460  94.704  -0.632   0.018   0.088   0.893
E S  1AHWC                 39    0.719  84.255  -1.059   0.005   0.015   0.979
E G  1AHWC                 40    0.660  51.934   0.203   0.018   0.019   0.964
E D  1AHWC                 41    0.569  81.964   1.217   0.018   0.088   0.893
B W  1AHWC                 42    0.129  30.928   0.860   0.252   0.423   0.325
E K  1AHWC                 43    0.368  75.718   1.346   0.478   0.309   0.214
E S  1AHWC                 44    0.594  69.629   1.118   0.662   0.225   0.113
E K  1AHWC                 45    0.330  67.799   1.275   0.649   0.163   0.188
B C  1AHWC                 46    0.041   5.799  -1.220   0.453   0.248   0.299
E F  1AHWC                 47    0.496  99.587   1.048   0.453   0.248   0.299
E Y  1AHWC                 48    0.499 106.572   0.229   0.199   0.152   0.649
B T  1AHWC                 49    0.161  22.372   0.698   0.113   0.087   0.800
E T  1AHWC                 50    0.501  69.433  -0.458   0.118   0.150   0.732
E D  1AHWC                 51    0.481  69.312   0.014   0.056   0.142   0.802
E T  1AHWC                 52    0.284  39.377   0.269   0.064   0.216   0.721
E E  1AHWC                 53    0.435  76.047  -0.147   0.069   0.386   0.545
B C  1AHWC                 54    0.037   5.139  -0.730   0.022   0.359   0.619
B D  1AHWC                 55    0.253  36.428  -0.565   0.021   0.451   0.528
B L  1AHWC                 56    0.055  10.034   0.531   0.066   0.296   0.638
B T  1AHWC                 57    0.149  20.722  -0.236   0.064   0.216   0.721
B D  1AHWC                 58    0.274  39.455  -0.705   0.199   0.152   0.649
E E  1AHWC                 59    0.446  77.916  -0.554   0.191   0.086   0.723
B I  1AHWC                 60    0.219  40.552  -0.981   0.199   0.152   0.649
E V  1AHWC                 61    0.355  54.548  -0.548   0.118   0.150   0.732
E K  1AHWC                 62    0.537 110.461  -0.640   0.113   0.087   0.800
E D  1AHWC                 63    0.464  66.834  -0.455   0.018   0.047   0.935
B V  1AHWC                 64    0.217  33.291   0.054   0.018   0.047   0.935
E K  1AHWC                 65    0.480  98.654  -0.925   0.004   0.138   0.858
B Q  1AHWC                 66    0.268  47.865  -0.534   0.005   0.336   0.660
E T  1AHWC                 67    0.278  38.545   1.212   0.003   0.718   0.279
B Y  1AHWC                 68    0.032   6.753   0.476   0.001   0.959   0.040
B L  1AHWC                 69    0.129  23.675   0.838   0.001   0.959   0.040
B A  1AHWC                 70    0.018   1.928   0.923   0.001   0.959   0.040
B R  1AHWC                 71    0.068  15.458   0.992   0.001   0.959   0.040
B V  1AHWC                 72    0.016   2.428   1.531   0.001   0.959   0.040
B F  1AHWC                 73    0.166  33.356   1.686   0.001   0.959   0.040
B S  1AHWC                 74    0.023   2.719   0.124   0.001   0.959   0.040
E Y  1AHWC                 75    0.266  56.930   0.726   0.001   0.900   0.099
E P  1AHWC                 76    0.297  42.088   0.321   0.004   0.514   0.481
E A  1AHWC                 77    0.502  55.298  -1.507   0.005   0.015   0.979
E G  1AHWC                 78    0.773  60.827  -1.329   0.003   0.003   0.994
E N  1AHWC                 79    0.491  71.897  -0.134   0.004   0.138   0.858
E E  1AHWC                 80    0.474  82.790  -0.253   0.005   0.336   0.660
B P  1AHWC                 81    0.134  18.986  -1.148   0.005   0.262   0.733
E L  1AHWC                 82    0.400  73.295  -0.361   0.021   0.279   0.699
B Y  1AHWC                 83    0.244  52.100  -1.066   0.021   0.451   0.528
E E  1AHWC                 84    0.339  59.311  -1.350   0.023   0.655   0.322
E N  1AHWC                 85    0.402  58.824  -0.574   0.023   0.655   0.322
B S  1AHWC                 86    0.166  19.490  -0.741   0.021   0.279   0.699
E P  1AHWC                 87    0.522  74.072  -0.699   0.020   0.205   0.775
E E  1AHWC                 88    0.454  79.349   0.209   0.022   0.359   0.619
B F  1AHWC                 89    0.072  14.410  -1.345   0.022   0.359   0.619
B T  1AHWC                 90    0.269  37.269   0.507   0.021   0.451   0.528
B P  1AHWC                 91    0.208  29.515  -0.629   0.021   0.451   0.528
B Y  1AHWC                 92    0.210  44.813   0.198   0.066   0.296   0.638
E L  1AHWC                 93    0.509  93.271  -0.749   0.064   0.216   0.721
E E  1AHWC                 94    0.578 100.959  -1.176   0.064   0.216   0.721
E T  1AHWC                 95    0.333  46.159  -1.134   0.052   0.084   0.864
E N  1AHWC                 96    0.530  77.577  -0.248   0.052   0.084   0.864
B L  1AHWC                 97    0.129  23.675  -1.588   0.018   0.088   0.893
E G  1AHWC                 98    0.357  28.127  -1.141   0.018   0.047   0.935
E Q  1AHWC                 99    0.342  61.063  -0.263   0.005   0.015   0.979
B P  1AHWC                100    0.161  22.860  -1.017   0.004   0.085   0.910
E T  1AHWC                101    0.523  72.568   0.273   0.004   0.514   0.481
B I  1AHWC                102    0.153  28.213  -0.583   0.001   0.900   0.099
E Q  1AHWC                103    0.478  85.353   0.634   0.001   0.959   0.040
B S  1AHWC                104    0.237  27.753  -0.236   0.001   0.959   0.040
E F  1AHWC                105    0.343  68.920   0.506   0.001   0.959   0.040
E E  1AHWC                106    0.393  68.710  -0.411   0.001   0.900   0.099
E Q  1AHWC                107    0.392  70.065  -0.134   0.004   0.616   0.381
E V  1AHWC                108    0.572  87.886  -0.774   0.005   0.262   0.733
E G  1AHWC                109    0.697  54.878  -0.802   0.005   0.015   0.979
E T  1AHWC                110    0.371  51.485  -0.019   0.004   0.138   0.858
B K  1AHWC                111    0.201  41.305   0.371   0.001   0.900   0.099
B V  1AHWC                112    0.025   3.842   0.559   0.001   0.959   0.040
B N  1AHWC                113    0.156  22.780   1.275   0.001   0.959   0.040
B V  1AHWC                114    0.020   3.074   1.006   0.001   0.959   0.040
B T  1AHWC                115    0.132  18.322   0.392   0.001   0.959   0.040
B V  1AHWC                116    0.027   4.135   0.533   0.001   0.900   0.099
E E  1AHWC                117    0.312  54.489   0.676   0.003   0.718   0.279
B D  1AHWC                118    0.151  21.745  -0.304   0.004   0.138   0.858
B E  1AHWC                119    0.118  20.615  -0.849   0.018   0.047   0.935
E R  1AHWC                120    0.341  77.997  -0.637   0.052   0.084   0.864
B T  1AHWC                121    0.294  40.736  -0.828   0.056   0.142   0.802
B L  1AHWC                122    0.208  38.121  -1.174   0.125   0.227   0.648
B V  1AHWC                123    0.236  36.289  -0.974   0.118   0.150   0.732
E R  1AHWC                124    0.480 109.920  -0.446   0.118   0.150   0.732
E R  1AHWC                125    0.612 140.171  -1.481   0.052   0.084   0.864
E N  1AHWC                126    0.668  97.737  -1.481   0.018   0.019   0.964
E N  1AHWC                127    0.652  95.482  -2.402   0.018   0.019   0.964
E T  1AHWC                128    0.643  89.226  -0.950   0.019   0.141   0.840
E F  1AHWC                129    0.458  91.840   0.142   0.021   0.279   0.699
B L  1AHWC                130    0.189  34.624   0.734   0.022   0.359   0.619
E S  1AHWC                131    0.309  36.203  -0.200   0.074   0.484   0.442
B L  1AHWC                132    0.118  21.569  -0.713   0.159   0.516   0.325
E R  1AHWC                133    0.406  92.997  -0.134   0.148   0.418   0.435
E D  1AHWC                134    0.401  57.799  -1.191   0.159   0.516   0.325
B V  1AHWC                135    0.198  30.402  -0.440   0.159   0.516   0.325
B F  1AHWC                136    0.215  43.211  -0.257   0.069   0.386   0.545
E G  1AHWC                137    0.481  37.823  -0.298   0.018   0.088   0.893
E K  1AHWC                138    0.569 117.064  -1.545   0.005   0.045   0.951
E D  1AHWC                139    0.567  81.719  -1.010   0.004   0.138   0.858
B L  1AHWC                140    0.097  17.816  -0.222   0.004   0.616   0.381
E I  1AHWC                141    0.284  52.522   0.796   0.001   0.900   0.099
B Y  1AHWC                142    0.043   9.125   0.600   0.001   0.959   0.040
B T  1AHWC                143    0.155  21.443   1.174   0.001   0.959   0.040
B L  1AHWC                144    0.018   3.259   1.309   0.000   0.983   0.017
B Y  1AHWC                145    0.124  26.477   1.233   0.000   0.983   0.017
B Y  1AHWC                146    0.033   7.116   0.292   0.000   0.983   0.017
B W  1AHWC                147    0.200  48.004   1.205   0.000   0.983   0.017
E K  1AHWC                148    0.317  65.166   0.766   0.001   0.900   0.099
E S  1AHWC                149    0.587  68.761  -0.402   0.005   0.262   0.733
E S  1AHWC                150    0.533  62.503  -0.541   0.005   0.045   0.951
E S  1AHWC                151    0.623  73.004  -0.298   0.005   0.045   0.951
E S  1AHWC                152    0.742  86.986   0.833   0.004   0.138   0.858
E G  1AHWC                153    0.363  28.576   0.425   0.004   0.514   0.481
E K  1AHWC                154    0.435  89.459   0.360   0.001   0.900   0.099
E K  1AHWC                155    0.433  89.171   0.734   0.001   0.959   0.040
E T  1AHWC                156    0.462  64.024   0.468   0.001   0.959   0.040
E A  1AHWC                157    0.360  39.650  -0.771   0.001   0.900   0.099
E K  1AHWC                158    0.402  82.712  -0.730   0.002   0.816   0.182
E T  1AHWC                159    0.608  84.343  -0.394   0.004   0.514   0.481
E N  1AHWC                160    0.586  85.732  -0.793   0.004   0.197   0.799
B T  1AHWC                161    0.250  34.689  -0.174   0.004   0.138   0.858
E N  1AHWC                162    0.400  58.560  -0.153   0.004   0.616   0.381
B E  1AHWC                163    0.232  40.478   0.286   0.001   0.900   0.099
E F  1AHWC                164    0.242  48.489  -0.043   0.001   0.900   0.099
B L  1AHWC                165    0.084  15.417   0.894   0.018   0.846   0.136
E I  1AHWC                166    0.427  78.976  -0.080   0.023   0.655   0.322
E D  1AHWC                167    0.468  67.410  -0.623   0.004   0.420   0.576
B V  1AHWC                168    0.092  14.187  -0.583   0.004   0.138   0.858
E D  1AHWC                169    0.581  83.708   0.208   0.005   0.045   0.951
E K  1AHWC                170    0.537 110.461  -0.603   0.005   0.015   0.979
E G  1AHWC                171    0.531  41.782  -1.324   0.005   0.015   0.979
E E  1AHWC                172    0.259  45.177  -0.166   0.004   0.138   0.858
E N  1AHWC                173    0.319  46.716  -0.243   0.003   0.718   0.279
B Y  1AHWC                174    0.057  12.266   1.158   0.001   0.900   0.099
B C  1AHWC                175    0.031   4.310   0.118   0.001   0.959   0.040
B F  1AHWC                176    0.032   6.382   0.627   0.001   0.959   0.040
B S  1AHWC                177    0.125  14.603   0.738   0.001   0.959   0.040
B V  1AHWC                178    0.020   3.028   1.260   0.001   0.959   0.040
B Q  1AHWC                179    0.153  27.397   0.942   0.001   0.959   0.040
B A  1AHWC                180    0.046   5.113  -0.774   0.001   0.900   0.099
B V  1AHWC                181    0.172  26.513   0.066   0.001   0.959   0.040
B I  1AHWC                182    0.071  13.227  -0.209   0.002   0.816   0.182
B P  1AHWC                183    0.214  30.338  -0.122   0.022   0.359   0.619
E S  1AHWC                184    0.268  31.398  -0.868   0.019   0.141   0.840
E R  1AHWC                185    0.432  98.905  -0.848   0.052   0.084   0.864
E T  1AHWC                186    0.476  66.007  -1.858   0.052   0.084   0.864
E V  1AHWC                187    0.430  66.060  -1.348   0.053   0.043   0.903
E N  1AHWC                188    0.577  84.531  -2.461   0.018   0.019   0.964
E R  1AHWC                189    0.418  95.814  -0.819   0.018   0.047   0.935
E K  1AHWC                190    0.496 101.945  -0.445   0.018   0.047   0.935
B S  1AHWC                191    0.186  21.846  -1.614   0.018   0.047   0.935
E T  1AHWC                192    0.563  78.046  -0.655   0.018   0.047   0.935
E D  1AHWC                193    0.447  64.413  -0.717   0.018   0.019   0.964
B S  1AHWC                194    0.149  17.463  -0.772   0.005   0.015   0.979
E P  1AHWC                195    0.546  77.406   0.954   0.018   0.019   0.964
E V  1AHWC                196    0.511  78.602   0.431   0.019   0.141   0.840
E E  1AHWC                197    0.433  75.645   1.575   0.022   0.552   0.426
B C  1AHWC                198    0.089  12.482  -1.731   0.023   0.655   0.322
E M  1AHWC                199    0.295  59.130   0.271   0.004   0.420   0.576
E G  1AHWC                200    0.819  64.455  -1.462   0.003   0.003   0.994

Explain the output.  Go back.
```

---
